# Associations of Clinical Stroke Misclassification (‘Clinical-Imaging Dissociation’) in Acute Ischemic Stroke

**DOI:** 10.1159/000286342

**Published:** 2010-02-19

**Authors:** Gillian Potter, Fergus Doubal, Caroline Jackson, Cathie Sudlow, Martin Dennis, Joanna Wardlaw

**Affiliations:** Department of Clinical Neurosciences, University of Edinburgh, Western General Hospital, Edinburgh, UK

**Keywords:** Acute ischemic stroke, Stroke subtype, Infarction, Acute stroke imaging, Diffusion-weighted imaging

## Abstract

**Background:**

Up to 20% of lacunar infarcts are clinically misdiagnosed as cortical infarcts and vice versa. The reasons for this discrepancy are unclear. We assessed clinical and imaging features which might explain this ‘clinical-imaging dissociation’ (C-ID).

**Methods:**

Patients with an acute stroke syndrome (cortical or lacunar) underwent magnetic resonance imaging including diffusion-weighted imaging (DWI). We recorded DWI-positive infarcts and proximity to cortex for small subcortical infarcts. We examined factors associated with C-ID.

**Results:**

137 patients with a mild cortical or lacunar syndrome had an acute ischemic lesion on DWI. Of these, 21/93 (23%) with a cortical syndrome had an acute lacunar infarct and 7/44 (16%) with a lacunar syndrome had an acute cortical infarct. From 72 patients with an acute lacunar infarct on DWI, lesion proximity to cortex (odds ratio (OR) 14.5, 95% confidence interval (CI) 1.61–130.1), left hemisphere location (OR 8.95, 95% CI 1.23–64.99) and diabetes (OR 17.1, 95% CI 1.49–196.16) predicted C-ID. On multivariate analysis of all 137 patients, C-ID was associated with diabetes (OR 7.12, 95% CI 1.86–27.2).

**Conclusions:**

C-ID occurs in a fifth of patients with mild stroke. Lacunar infarcts lying close to cortex are more likely to cause cortical symptoms. Diabetes is associated with any clinical-imaging mismatch. Stroke misclassification which can arise with clinical classification alone should be minimized in research by verification with high-sensitivity imaging.

## Introduction

Classification of acute ischemic stroke subtypes is important for categorizing patients into aetiologic and prognostic subgroups in clinical trials, epidemiological and pathophysiological studies, and may help guide patient management in clinical practice [1]. Although it is well established that infarcts in particular locations are associated with specific clinical symptoms, a proportion of patients with acute ischemic stroke will be incorrectly subtyped based on clinical assessment alone.

The Oxford Community Stroke Project (OCSP) clinical classification categorizes patients based on clinical assessment alone into those with a lacunar syndrome (LACS), a partial anterior circulation syndrome (PACS), a total anterior circulation syndrome (TACS) or a posterior circulation syndrome (POCS) [2]. PACS and TACS both indicate cortical stroke syndromes. The OCSP classification can predict correctly the site and size of cerebral infarct, if visible, on computed tomography (CT) or magnetic resonance (MR) brain imaging in about three quarters of patients [3]. However, numerous studies demonstrate that about a fifth of patients with a cortical syndrome clinically have an acute lacunar infarct on imaging that accounts for their recent stroke symptoms; similarly some patients with LACS clinically have an acute cortical infarct on brain imaging that accounts for their recent stroke symptoms [4,5,6,7,8,9,10,11,12,13,14,15], creating a ‘clinical-imaging dissociation’ (C-ID) (table 1). This dissociation is important because epidemiological studies, primary treatment and secondary prevention trials in stroke have so far relied heavily on clinical classification so are likely to have incorporated ‘noise’ due to approximately one fifth of LACS and PACS patients being misclassified.

Several factors may contribute to C-ID. Previous studies suggested that the side of brain affected by stroke [3], leukoaraiosis [4], clinical severity [4,13] and asymptomatic infarcts on imaging [4] were associated with C-ID. Other contributing factors have not been assessed. Delays in clinical assessment may allow neurological signs to resolve making an accurate history difficult to obtain (e.g. dysarthria and dysphasia can be difficult to distinguish on history alone); clinical examination may be insensitive to some subtle cortical signs (e.g. mild inattention) which would distinguish PACS from LACS. However, the one previous study that examined delay to diagnosis did not find any association with C-ID [3]. Reliability of classification is affected by observer expertise in use of the OCSP classification, particularly in minor stroke [16]. C-ID may also arise when there is failure of brain imaging to ascertain relevant ischemic lesions, either because the imaging is relatively insensitive to small acute lesions [17] or is performed too late to identify the acute lesion reliably.

**Fig. 1:**
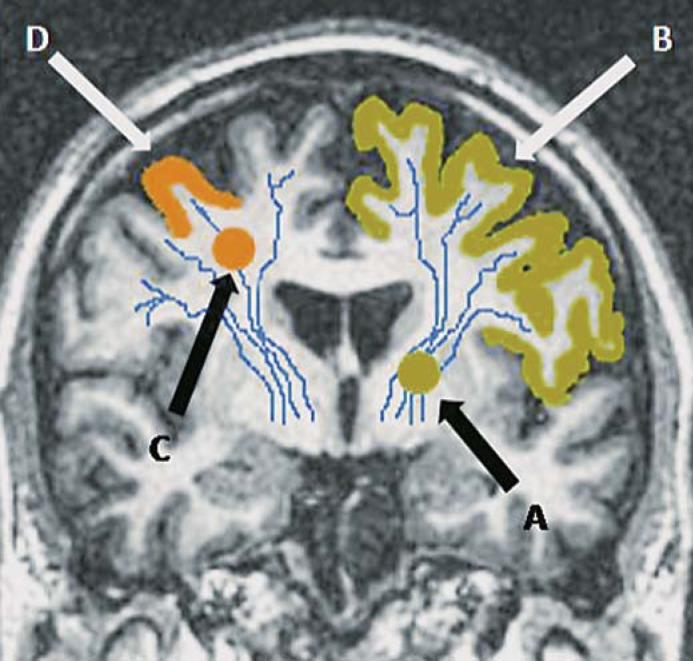
Coronal T_1_-weighted MRI brain to demonstrate how the site of a small subcortical (lacunar) infarct could influence clinical presentation. A small subcortical infarct lying in the left internal capsule, i.e. deep white matter (A), would cause functional disconnection of a large area of cortex (B, shaded). A peripheral small subcortical infarct lying close to cortex (C) would affect only a limited area of cortex (D, shaded), and could mimic a mild cortical stroke.

Small subcortical infarcts are considered to cause their symptoms because their location in the subcortical white matter or basal ganglia effectively disconnects a larger section of cortex than is affected by an equivalent-sized lesion in the cortex (fig. 1). We hypothesized that a small subcortical infarct lying close to the cerebral cortex could mimic symptoms of a mild cortical syndrome (PACS) by causing functional disconnection of only a small area of cortex compared to one of the same size lying in the periventricular white matter or basal ganglia which would disconnect a larger area of cortex. We also considered that other factors, such as previous stroke, could increase the proportion with C-ID, as residual features of a previous stroke could make interpretation of features due to the acute stroke difficult, and that common stroke risk factors might influence symptomatology.

## Methods

We included patients from a prospectively collected hospital-based stroke register of consecutive stroke and transient ischemic attack (TIA) patients seen at a large academic teaching hospital between April 2002 and May 2005. In the present study we included only those patients who underwent brain MR imaging (MRI). We performed MR when time from stroke onset was greater than 5–7 days or uncertain, if there was clinical uncertainty about the definite diagnosis of stroke (particularly in patients with prior stroke) or of the vascular territory involved (carotid or vertebrobasilar), if there was a potential underlying cause of stroke that required further investigation by advanced brain imaging, or if the patient was suitable for inclusion into other studies of large artery or subcortical stroke requiring brain MRI.

All patients were assessed by an experienced stroke physician who took a detailed history, performed a general and neurological examination and recorded the National Institutes of Health Stroke Scale score. Patients were assigned a clinical subtype according to the OCSP classification based on the maximum stroke deficit as described previously [1]. Lacunar and mild cortical syndromes (LACS and PACS, respectively) were defined according to the OCSP classification [1]. LACS was defined as one of the classical LACSs – i.e. pure motor weakness and/or sensory loss of face and arm, arm and leg or all three, or ataxic hemiparesis (ipsilateral corticospinal and cerebellar-like dysfunction without other features clearly localizing to the posterior circulation, including dysarthria-clumsy hand syndrome and homolateral ataxia and crural paresis) – in the absence of visual field defect or higher cerebral dysfunction. In patients with faciobrachial or brachiocrural motor and/or sensory deficits, only involvement of the whole limb was considered acceptable for LACS; patients with involvement of less than the whole limb were classified as PACS. Mild cortical stroke syndrome (PACS) was defined as a maximum clinical deficit of either: weakness or sensory loss in the face, arm or leg; loss of higher cerebral function (e.g. dysphasia or neglect); or weakness in more than one limb in the presence of loss of higher cerebral dysfunction or homonymous hemianopia. Isolated homonymous hemianopia was classified as POCS [1], a relatively crude grouping of posterior circulation cortical and lacunar lesions with clinical consequences which are generally less predictable because of the greater frequency of developmental vascular anomalies and greater variability of the territory supplied by individual arteries.

All patients had MRI including diffusion-weighted imaging (DWI), carotid Doppler ultrasound, electrocardiogram, blood tests, and other investigations as indicated. We recorded risk factors including diabetes mellitus (defined as having a previous diagnosis of, or being on current medication for, diabetes), hypertension (defined as having a history of hypertension requiring medication) and prior history of stroke (i.e. clinical presentation with stroke). The Edinburgh Stroke Study was approved by the Local Research Ethics Committee and all patients (or their relatives) gave written informed consent. Patients underwent 1.5 T MRI (GE Signa LX EchoSpeed scanner, Milwaukee, Wisc., USA). We collected sets of axial diffusion-weighted echo planar (EP) images (sensitization levels b = 0 and 1,000 s/mm^2^) with 5-mm slice thickness, 1-mm slice gap, 128 × 128 image matrix and 24 × 24 field of view. Other MR parameters have been published elsewhere [18]. Images were reviewed by a neuroradiologist (G.P.), blinded to all clinical details. Location and size of recent infarcts were recorded. Recent infarcts were defined as hyperintense on DWI, hypointense on the apparent diffusion coefficient map and either normal or hyperintense to normal brain on fluid-attenuation inversion recovery (FLAIR)/T_2_-weighted imaging (less hyperintense than cerebrospinal fluid on T_2_). Lacunar infarcts were defined as round or ovoid lesions measuring ≤20 mm in maximal diameter in the white matter, basal ganglia or brainstem. Proximity to cortex of recent lacunar infarcts was noted on any sequence on which the infarct was visible. We defined ‘close to cortex’ as the edge of the infarct lying within 2 mm of the cortical margin in the white matter. Infarcts were defined as cortical where there was a typical configuration with involvement of cortex ± adjacent white matter, and striatocapsular where located in the basal ganglia or centrum semiovale and measuring ≥20 mm. Uncertain lesions were checked with a second neuroradiologist (J.W.). A lacunar infarct was considered ‘appropriate’ in patients presenting with LACS; a lacunar infarct in the brainstem or thalamus, i.e. in the vertebrobasilar territory, was also considered ‘appropriate’ to POCS. A small- or medium-sized cortical infarct was considered ‘appropriate’ in PACS. We also recorded white matter hyperintensities (WMH) (0–3 on the Fazekas scale [19]); old strokes using all sequences; enlarged perivascular spaces (EPVS, defined as ≤2 mm round or linear isointense to cerebrospinal fluid lesions along the course of penetrating arteries, T_2_-hyperintense and T_1_/FLAIR-hypointense) in the basal ganglia and centrum semiovale (0–4 on a local scale, where 0 = none and 4 = >40) [20] and atrophy (0–3 on a validated scale, where 0 = none and 3 = severe) [21].

### Statistical Analysis

We assessed the statistical significance of differences in baseline characteristics and brain-imaging features using Student's t test for continuous variables, the Mann-Whitney test for non-normally distributed continuous variables, and the χ^2^ test for dichotomous variables. We performed multivariable analyses using logistic regression to determine independent factors for C-ID. In the logistic regression model we included all variables from univariate analysis and obtained adjusted odds ratios (OR) (comparing patients with C-ID versus those without) and 95% confidence intervals (CIs). We dichotomized scores for WMH (0–1 vs. 2–3), brain tissue loss (0–1 vs. 2–3) and EPVS (0–1 vs. 2–4) due to low frequencies. We performed analyses with Minitab Statistical Software Version 15 (Minitab, Inc., State College, Pa., USA).

## Results

**Fig. 2:**
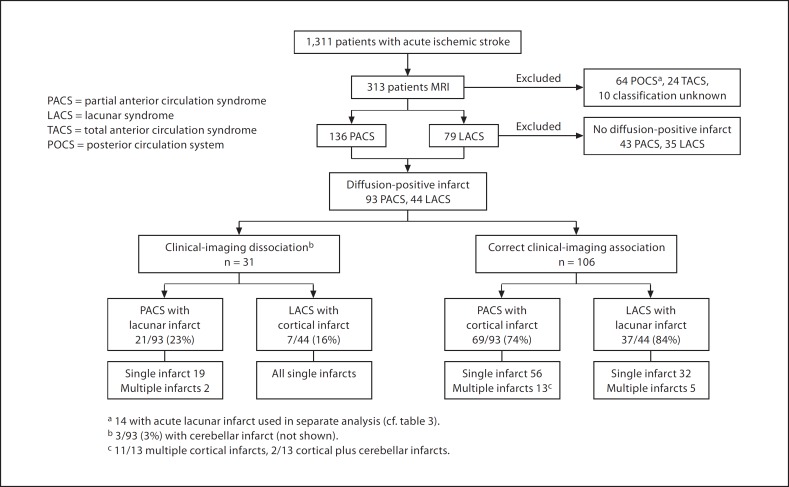
Identification of patients with PACS and LACS for assessment of C-ID and imaging findings.

Amongst the 1,311 acute ischemic stroke patients recruited to the Edinburgh Stroke Study, 313 underwent MR brain imaging, of whom 136 (43%) presented clinically with PACS, 79 (25%) with LACS, 24 (8%) with TACS, 64 (21%) with POCS, and 10 (3%) with an uncertain OCSP classification (fig. 2). Ninety-three (68%) of 136 patients with PACS and 44/79 (56%) patients with LACS had a diffusion-positive infarct relevant to the clinical presentation (fig. 2). Six (4%) patients with PACS and 3 (4%) LACS in whom DWI was normal had lesions on other sequences as the likely cause of symptoms. Patients undergoing MRI were slightly younger when compared with the 1,311 ischemic stroke patients from which we identified our study population, with a higher proportion of males and a lower prevalence of diabetes but the proportion of PACS and LACS were similar (online suppl. table 2, www.karger.com/doi=10.1159/000286342).

Sixty-nine (74%) patients presenting with PACS had an acute cortical infarct (all small- or medium-sized and considered appropriate to clinical syndrome) (fig. 2), 21 (23%) had an acute lacunar infarct (fig. 2), and 3 (3%) had a cerebellar infarct on DWI. Of patients presenting with LACS, 37/44 (84%) had an acute lacunar infarct and 7 (16%) had an acute cortical infarct. Most acute lacunar infarcts identified on DWI in patients presenting with LACS or PACS were located in the centrum semiovale (18/37 LACS, 20/21 PACS). Amongst patients with an acute infarct on DWI, 65/138 (47%) patients had old infarcts on imaging, the median WMH score was 1.62 (range 0–3) and the median EPVS score was 2 (range 0–4).

In the cohort of 137 patients with PACS and LACS and an acute infarct on DWI, C-ID was associated on univariate analysis with diabetes (p = 0.001), increasing time from onset of stroke symptoms to MRI (p = 0.05), EPVS (p = 0.02) and old stroke lesions on brain imaging (p = 0.02), but not with age (p = 0.69), history of previous stroke (p = 0.08), brain tissue loss (p = 1.0) or WMH (0.12; table 2). On multivariate analysis, diabetes (OR 7.12, 95% CI 1.86–27.2; p = 0.004) was independently associated with C-ID.

Multiple acute infarcts were not associated with C-ID either: amongst 44 LACS, 5 (11%) had multiple DWI-positive infarcts (all lacunar lesions) and none had C-ID. Amongst 93 PACS, 15 (16%) had multiple DWI-positive infarcts, of whom 2 (13%) had C-ID (all multiple lacunar infarcts) and 13 (87%) were correctly associated (showing multiple cortical, or cortical plus cerebellar, infarcts; fig. 2).

We examined characteristics associated with a lacunar infarct causing PACS clinical syndrome in all 72 patients with an acute lacunar infarct on DWI. Thirty-seven (51%) patients presented with LACS, 21 (29%) with PACS, and 14 (20%) with POCS (fig. 2). Lacunar infarcts in POCS patients were located (appropriate to symptoms) in the posterior circulation territory (12 brainstem, 1 thalamus, 1 posterior border zone) and were therefore not considered to have C-ID. C-ID was associated in univariate analyses with increasing age (p = 0.03), hypertension (0.004), increasing delay from symptom onset to clinical examination (p = 0.001) and to MRI (p = 0.04) and infarct positioned close to cortex (p = 0.001) (table 3). In multivariate analysis, closeness to cortex (OR 14.5, 95% CI 1.61–130.1; p = 0.02) and older age (OR 1.16, 95% CI 1.0–1.30; p = 0.01) remained independently associated with C-ID; diabetes (OR 17.1, 95% CI 1.49–195.16; p = 0.02) and left-hemispheric location (OR 8.95, 95% CI 1.23–64.99; p = 0.03) were also independent associates. There was no difference in the size of the lacunar infarcts between those causing PACS and those causing LACS clinical syndromes (mean 11.7 ± 3.4 vs. 10.8 ± 4.3 mm; p = 0.32; table 3), nor in the size of those lacunar infarcts that were close to cortex and caused PACS (n = 16) or LACS (n = 15) (mean 12.3 ± 5.3 vs. 12 ± 3.7 mm; p = 0.8).

## Discussion

In our study of acute stroke patients with PACS and LACS and an acute infarct on DWI, we found that 23% of patients presenting with PACS had an acute lacunar infarct, and 16% of patients presenting with LACS had an acute cortical infarct and no other explanation for their recent stroke symptoms. The main factors associated with C-ID amongst all patients in this study, after adjusting for potential confounders, was diabetes (old stroke lesions and previous history of stroke were associated in univariate analysis only), and in patients with an acute lacunar infarct on imaging, proximity of the lacunar infarct to the cortex, older age, diabetes and left hemisphere location. Lesion size, multiple acute infarcts, time to scanning, WMH, brain atrophy and history of prior stroke were not associated with C-ID.

The present study has some methodologic strengths. We performed a more comprehensive examination of associated features than in previous studies of C-ID. We identified consecutive stroke and TIA patients presenting to our stroke service. Patients undergoing MRI had similar proportions of PACS and LACS to the registry cohort as a whole. The minor differences between patients undergoing MRI and those that did not (slightly younger, more males, fewer with vascular risk factors) is unlikely to have influenced the generalizability of our results. All patients were very carefully examined by an experienced stroke physician and categorized according to strict interpretation of the OCSP criteria. Images were reviewed systematically according to a structured proforma by a trained rater using validated scales.

There are limitations of our study. Overall, only 313/1,311 (24%) of patients presenting with acute stroke underwent MRI, which may have introduced a selection bias. Other factors which may have led to selection bias were the inclusion of patients with increasing delay, or uncertain time, since stroke onset, and where there was clinical uncertainty about stroke diagnosis. Our sample may therefore have included an overrepresentation of patients who were more difficult to subtype. However, this does not negate the observation that lacunar lesion location was associated with C-ID. Median time from stroke onset to MRI of 19 days (many were outpatients, with mild stroke), i.e. outside the time period generally considered optimal for DWI, and only 64% (137/215) patients had a diffusion-positive infarct. However, a previous study showed no difference in the proportion with an acute infarct on DWI in those scanned before versus after 4 weeks [22]. Although several patients had DWI outside the optimal time period, previous work has shown that DWI may also be useful up to several weeks after stroke onset [23]. We also cannot exclude the possibility that the infarct responsible for initial symptoms was no longer visible on DWI by the time the patient underwent brain imaging, and that a new, silent DWI infarct (but sufficiently consistent with the infarct location as suggested by the symptoms and signs as to be considered as the acute index infarct) had appeared in this period, but this possibility was considered to be low and consequently not a significant confounding factor. We did not investigate underlying mechanisms as a cause of C-ID, and the study was not designed to test the effect of clinician experience on misdiagnosis, a factor identified in one previous study [16].

Previous studies did not consider proximity of lacunar infarcts to cortex or diabetes as possible factors for C-ID. The association with diabetes may be partly explained by a co-association with old ‘silent’ infarcts; however, although old infarcts were associated with C-ID in univariate analysis, they did not remain independently associated in multivariate analysis, as found previously [4]. Assigning clinical subtype may be more difficult in the presence of an old infarct, even if clinically silent, as signs from the previous infarct may confuse the clinical picture. The association of left hemisphere location and C-ID is consistent with previous studies which found that left-sided lesions were more common in patients with PACS/non-LACS with C-ID [4] and that right-sided lesions were more common in LACS presenting with C-ID [3]. This may be explained by the difficulty in distinguishing dysarthria from dysphasia, especially if symptoms and signs were mild or had resolved by the time of assessment. We did not find an association between C-ID and WMH, in contrast to one previous study [4], possibly because the latter used CT, which is less sensitive to WMH than MR FLAIR or T_2_. We found that 11% of patients with LACS had multiple acute lacunar infarcts, similar to previous studies [11,14,15], but multiplicity of infarcts did not contribute to C-ID. We did not find an association between time lapse from stroke onset to MRI and C-ID, in agreement with one previous study using CT [3]. Ability to recall symptoms and signs may deteriorate with increasing time to assessment, particularly with speech disorders. Others have reported problems with very early or very late diagnosis of lacunar stroke [24]. Our use of maximum deficit in assigning the OCSP subtype may have overcome any effect of time on stroke diagnosis.

C-ID has important implications for research into epidemiology, pathophysiology and treatment of lacunar stroke as well as for clinical practice. In research which relies heavily on clinical presentation alone, results may be affected by ‘noise’ caused by C-ID of between 10 and 20% patients with mild stroke. Studies in which CT is used in conjunction with clinical classification will also be affected, since CT is less sensitive than DWI for small acute infarcts [17], particularly when performed soon after symptom onset, as is increasingly the case. Acute ischemic stroke lesions are visible on CT in <20% of LACS and <35% PACS (i.e. mild stroke) at <3 h, rising to approximately 45 and 60% at 36 h [25]. The debate over mechanisms of lacunar stroke – up to 20% are said to be associated with cardiac and large artery atherothromboembolism [26,27] rather than intrinsic small vessel disease – could be explained by C-ID. Similarly, large primary and secondary prevention trials of ischemic stroke testing aspirin, cholesterol-lowering drugs and antihypertensives have relied heavily on clinical classification and CT [28]. ‘Noise’ from C-ID may have impeded the demonstration of any difference in treatment effects between stroke subtypes, if one existed. In future, where precise diagnosis of stroke subtype and lesion location is important, lesion location should be verified by sensitive imaging.

## Figures and Tables

**Table 1:** Previous studies identifying clinical-imaging dissociation (C-ID), and features examined

Reference	Setting	Brain imaging	Ischemic stroke subtype classification	LACS	LACS/large subcortical or cortical infarct (%)	Cortical syndrome	Cortical syndrome/lacunar infarct (%)	Features examined in relation to C-ID
Lodder, 1994 [4]	Hospital	CT	−	147	23 (16)	203	19 (9)	Disability (OR 4.31, 95% CI 1.25–14.88) Leukoaraiosis (non-LACS; OR 3.79, 95% CI 1.32–10.05)
								Asymptomatic infarcts (non-LACS; OR 4.13, 95% CI 1.45–11.71) Hemisphere affected (non-LACS)

Al-Buhairi, 1998 [5]	Hospital	CT	OCSP	–	–	121	4 (5)	–

Pittock, 2003 [6]	Hospital	CT	OCSP	47	2 (10)	24	3 (11)	–

Wlodek, 2004 [7]	Hospital	CT	OCSP	101	29 (29)	193	29 (15)	–

Kobayashi, 2009 [8]	Hospital	CT	OCSP	60	19 (31)	183	3 (2)	–

Mead, 1999 [9]	Hospital	CT, MRI	OCSP	180	35 (19)	395	62 (16)	–

Mead, 2000 [3]	Hospital	CT, MRI	OCSP	144	35 (24)	298	38 (13)	Hemisphere affected (PACS, LACS)

Anderson, 2004 [10]	Hospital, community	CT, MRI	OCSP	69	12 (17)	75	16 (21)	–

Ay, 1999 [11]	Hospital	DWI	–	62	1 (2)	–	–	–

Lindgren, 2000 [12]	Hospital	DWI	–	23	2 (9)	–	–	–

Allder, 2003 [13]	Hospital	DWI	OCSP	–	–	42	6 (14)	Clinical severity (χ^2^ 18.9, p < 0.01)

Seifert, 2005 [14]	Hospital	DWI	OCSP	–	–	93^a^	14 (15)	–

Wessels, 2005 [15]	Hospital	DWI	–	73	13 (18)^b^	–	–	–

This study	Hospital	DWI	OCSP	80	7 (16)	136	24 (25)	Old infarcts (OR 3.02, 95% CI 1.06–8.59) Diabetes (OR 7.17, 95% CI 1.86–27.71)

PACS = Partial anterior circulation syndrome; LACS = lacunar syndrome; OCSP = Oxford Community Stroke Project; OR = odds ratio; CI = confidence interval; DWI = diffusion-weighted imaging; CT = computed tomography; MRI = magnetic resonance imaging.

a Patients with subcortical or brainstem lesions <1.5 cm in diameter.

b Four with single cortical lesion, 9 with scattered or multiple lesions containing a cortical lesion.

**Table 2:** Factors associated with clinical-imaging dissociation (C-ID) in patients with PACS and LACS and an acute infarct on DWI

	C-ID (n = 31)	No C-ID (n = 106)	Univariate statistic and test score	Univariate p value	Multivariate p value	Multivariate OR (95% CI)
Demographics						
Age, years	71 ± 11	70 ±13	Student's t test −0.4	0.69	0.1	1.04 (0.98–1.10)^1^
Gender, male	19 (61)	64 (60)	χ^2^ 0.008	0.93	0.74	1.21 (0.40–3.70)
Medical history						
Previous stroke, %	12 (39)	24 (23)	χ^2^ 3.03	0.08	0.14	2.38 (0.74–7.60)
Hypertension, %	23 (74)	58 (56)	χ^2^ 3.52	0.06	0.88	1.09 (0.36–3.34)
Diabetes, %	9 (29)	6 (6)	χ^2^ 11.2	0.001	0.004	7.12 (1.86–27.2)
Clinical						
Median days, onset to assessment	16	11	Mann-Whitney 5 (0–12)	0.09	0.72	1.01 (0.96–1.07)
Range (IQR)	0–97 (10–23)	0–125 (1–22)				
Median days, onset to MRI	21	15	Mann-Whitney 8 (0–14)	0.05	0.71	1.01 (0.9–1.06)
Range (IQR)	0–97 (14–33)	0–140 (1–31)				
MR brain imaging characteristics						
Left hemisphere, %	17 (55)	61 (58)	χ^2^ 0.07	0.79	0.49	1.44 (0.51–4.09)
WMH 2–3, %^a^	16 (53)	39 (37)	χ^2^ 2.62	0.12	0.85	1.12 (0.35–3.55)
EPVS 2–4, %^b^	19 (63)	41 (340)	χ^2^ 5.2	0.02	0.38	1.61 (0.56–4.60)
Brain tissue loss 2–3, %^c^	8 (28)	29 (28)	χ^2^ <0.001	1.0	0.1	0.33 (0.09–1.24)
Old stroke lesions, %	20 (65)	45 (42)	χ^2^ 5.49	0.02	0.08	2.56 (0.89–7.36)

EPVS = Enlarged perivascular spaces; WMH = white matter hyperintensities; IQR = interquartile range; OR = odds ratio; CI = confidence interval.

1 Odds ratio (OR) per additional year of age.

a On Fazekas scale.

b On EPVS scale.

c On brain tissue loss scale.

**Table 3:** Associations with clinical-imaging dissociation (C-ID) in all subjects with an acute lacunar infarct on DWI (n = 72)

	C-ID (n = 22)	No C-ID (n = 50)	Univariate statistic and test score	Univariate p value	Multivariate p value	Multivariate OR (95% CI)
Demographics						
Age, years	75 ±10	69 ± 11	Student's t test −2.33	0.03	0.01	1.16 (1.03–1.30)^1^
Gender, male (%)	14 (64)	27 (54)	χ^2^ 0.58	0.45	0.09	5.25 (0.78–35.41)
Medical history						
Previous stroke, %	6 (27)	8 (16)	χ^2^ 1.24	0.27	0.64	1.66 (0.20–13.82)
Hypertension, %	18 (82)	22 (44)	χ^2^ 8.85	0.004	0.12	3.72 (0.72–19.28)
Diabetes, %	6 (27)	4 (8)	χ^2^ 4.75	0.06	0.02	17.1 (1.49–195.16)
Clinical						
Median days, onset to assessment	18	14	Mann-Whitney −6	0.04	0.49	1.05 (0.91–1.21)
Range (IQR)	1–97 (14–23)	0–134 (3–22)				
Median days, onset to MRI	27	20	Mann-Whitney −6	0.04	0.76	0.98 (0.84–1.13)
Range (IQR)	6–97 (16–32)	0–141 (6–29)				
MR brain imaging characteristics						
Subcortical infarct close to cortex, %	16 (73)	15 (30)	χ^2^ 11.4	0.001	0.02	14.5 (1.61–130.1)
Infarct size, mm	11.7±3.4	10.8±4.3	Student's t test −1.01	0.32	0.99	1.00 (0.82–1.23)
Left hemisphere location, %	15 (68)	26 (52)	χ^2^ 1.22	0.27	0.03	8.95 (1.23–64.99)
WMH 2–3, %^a^	12 (55)	20 (40)	χ^2^ 1.31	0.25	0.43	0.48 (0.08–2.93)
EPVS 2–4, %^b^	14 (64)	25 (49)	χ^2^ 0.98	0.32	0.67	1.52 (0.22–10.46)
Brain tissue loss 2–3, %^c^	7 (32)	9 (18)	χ^2^ 1.69	0.19	0.35	0.30 (0.02–3.75)
Old stroke lesions, %	14 (64)	23 (46)	χ^2^ 1.9	0.17	0.13	0.20 (0.02–1.65)

EPVS = Enlarged perivascular spaces; WMH = white matter hyperintensities; IQR = interquartile range; OR = odds ratio; CI = confidence interval.

1 Odds ratio (OR) per additional year of age.

a On Fazekas scale.

b On EPVS scale.

c On brain tissue loss scale.
